# Constructing higher-order miRNA-mRNA interaction networks in prostate cancer via hypergraph-based learning

**DOI:** 10.1186/1752-0509-7-47

**Published:** 2013-06-19

**Authors:** Soo-Jin Kim, Jung-Woo Ha, Byoung-Tak Zhang

**Affiliations:** 1 Interdisciplinary Program in Bioinformatics, Seoul National University, Seoul 151-742, Korea; 2 Center for Biointelligence Technology (CBIT), Seoul National University, Seoul 151-742, Korea; 3 School of Computer Science and Engineering, Seoul National University, Seoul 151-742, Korea

**Keywords:** miRNA-mRNA interaction networks, Hypergraph-based model, Higher-order gene modules, Evolutionary learning, Cancer genomics data analysis

## Abstract

**Background:**

Dysregulation of genetic factors such as microRNAs (miRNAs) and mRNAs has been widely shown to be associated with cancer progression and development. In particular, miRNAs and mRNAs cooperate to affect biological processes, including tumorigenesis. The complexity of miRNA-mRNA interactions presents a major barrier to identifying their co-regulatory roles and functional effects. Thus, by computationally modeling these complex relationships, it may be possible to infer the gene interaction networks underlying complicated biological processes.

**Results:**

We propose a data-driven, hypergraph structural method for constructing higher-order miRNA-mRNA interaction networks from cancer genomic profiles. The proposed model explicitly characterizes higher-order relationships among genetic factors, from which cooperative gene activities in biological processes may be identified. The proposed model is learned by iteration of structure and parameter learning. The structure learning efficiently constructs a hypergraph structure by generating putative hyperedges representing complex miRNA-mRNA modules. It adopts an evolutionary method based on information-theoretic criteria. In the parameter learning phase, the constructed hypergraph is refined by updating the hyperedge weights using the gradient descent method. From the model, we produce biologically relevant higher-order interaction networks showing the properties of primary and metastatic prostate cancer, as candidates of potential miRNA-mRNA regulatory circuits.

**Conclusions:**

Our approach focuses on potential cancer-specific interactions reflecting higher-order relationships between miRNAs and mRNAs from expression profiles. The constructed miRNA-mRNA interaction networks show oncogenic or tumor suppression characteristics, which are known to be directly associated with prostate cancer progression. Therefore, the hypergraph-based model can assist hypothesis formulation for the molecular pathogenesis of cancer.

## Background

Prostate cancer is a common disease in the male population, induced by complex interactions among various genetic factors [1]. As such, the pathological causes of this disease are not easily identified. Recent human cancer studies have demonstrated that most cancer regulations are related to modular construction and combinatorial control by multiple genetic factors. This module-based view of higher-order relationships can provide new insights into the behavior of complex biological systems [2,3].

Recently, miRNAs have caused great excitement as diagnostic and therapeutic signatures of prostate cancer [4-8]. They play important roles in cancer pathogenesis, including disease onset, progression, and metastasis, by regulating the stability and translation efficiency of their target mRNAs. Thus, the functional relationships between miRNAs and mRNAs should be elucidated to identify key transcriptional circuits involved in cancer regulation. However, analyzing higher-order miRNA-mRNA relationships is rendered as a challenging problem due to the complexity of their interactions.

Modern cancer research has progressed from identifying biomarkers to systemically exploring gene interactions [9-11]. Many studies have focused on the interaction of genetic components at the systems level. Computational methods, which analyze gene regulatory interactions on a genome-wide scale from high-throughput biological data, have flourished in recent decades [12-14]. In addition, systems biology has proposed to build miRNA regulation networks underlying the development of many human diseases [15-17]. Moreover, miRNA regulatory mechanisms are now thought to be inferable from miRNA-mRNA interactions [18-20]. Several studies have attempted to identify groups of coherent miRNAs and mRNAs that cooperate in biological processes from heterogeneous data sources via various computational approaches, including probabilistic methods [21-28], rule-based learning [29,30], matrix factorization [31], and statistical methods [32-35]. These approaches have simplified complex biological mechanisms by systematically analyzing the relationships between genetic elements at the genome level. Typically, however, bi-relationships between only two factors are assumed in many previous studies [21,30-35]. Such restrictions are unsuitable for complex genetic interactions because information is lost under the assumption, and biological regulation is controlled by the interaction of multiple genetic components. Many studies have also investigated miRNA-mRNA regulatory interactions using biological information, especially miRNA-target information [21-25,29-33]. Biological information reduces the number of false positives, since it provides the predictive model with prior knowledge. In contrast, unknown or hidden interactions not involved in the prior knowledge may be difficult to identify from this information. To avoid this problem, some probabilistic models which infer miRNA-mRNA modules from expression profiles only, without relying on target information, have been proposed [26-28]. Bonnet’s model, called LeMoNe [26,27], consists of two major steps; the generation of gene clusters based on a feature-sample co-clustering method, and the inference of regulatory modules from generated clusters and regulators based on probabilistically optimized trees. In the clustering approach of Bonnet’s method, gene regulatory modules underlying a specific cancer stage are not easily identified. Liu’s approach infers functional miRNA regulatory modules using Correspondence Latent Dirichlet Allocation (Corr-LDA) [28]. The Corr-LDA based model requires discretized data. Since the Corr-LDA model infers probability distributions from latent variables, moreover, miRNAs can be annotated to any functional modules, while mRNAs are restricted to the miRNA-inferred modules.

**Figure 1:**
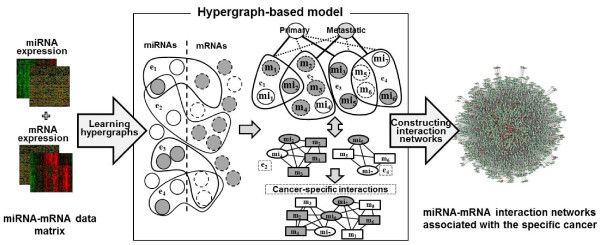
**Overview of the hypergraph-based model for constructing higher-order miRNA-mRNA interaction networks at a specific cancer stage.** Solid and dotted circles denote miRNAs and mRNAs, respectively. Closed curves denote hyperedges (i.e. modules). In the conventional graph representation (two graphs in the right-bottom of the central box of the figure), ellipses and boxes denote miRNAs and mRNAs, respectively. Grey and white indicate respective high and low gene expression levels.

Here we introduce a data-driven model for identifying cancer stage-specific interactions that reflects the high-order relationships between miRNAs and mRNAs (Figure 1). The proposed model is a hypergraph comprising numerous hyperedges, representing the multi-variable combinations corresponding to miRNAs and mRNAs. Each hyperedge is formally defined as cancer-stage specific statistical figures, and thus our model can deal with real-valued data without discretization. The weight of a hyperedge reflects the strength of the higher-order dependency among the variables of the hyperedge. Therefore, each hyperedge potentially behaves as a gene module. The model explicitly constructs a complex interaction network from many such gene modules. The model is learned by finding a highly-discriminate hypergraph structure from expression profiles using data relevant to a certain stage of prostate cancer.

The learning process involves the iteration of two learning phases; structure and parameter. The structure learning phase constructs a hypergraph of putative hyperedges for discovering potential gene interactions, from a huge feature space represented by the combinations of many miRNAs and mRNAs. Because the miRNA-mRNA interactions are intractably complex, we adopt an evolutionary strategy based on an information theoretic co-regulatory measure, called mutual information. This strategy is used to select genetic variables for generating hyperedges. During the parameter learning phase, the hypergraph is refined by updating the weights of the hyperedges (representing higher-order miRNA-mRNA modules). To this end, we employ a gradient descent method similar to the back-propagation algorithm for learning artificial neural networks. The learned model is then converted into a network structure reflecting the cooperative higher-order gene activities by connecting the extracted hyperedges. Data-driven learning allows the model to build new miRNA-mRNA interaction networks which display the hidden properties of primary and metastatic prostate cancers from a given dataset, which are not known *a priori*.

We construct cancer stage-specific miRNA-mRNA interaction networks reflecting their higher-order relationships using the MSKCC Prostate Oncogenome Project dataset [36] from the model. We demonstrate that the proposed model can build several biologically significant miRNA-mRNA interaction networks, including potential modules associated with primary and metastatic prostate cancer. Moreover, cancer-related miRNAs and genes dominate the identified interactions. Some of these interactions, such as hsa-miR-1, hsa-miR-133a, hsa-miR-143, hsa-miR-145, hsa-miR-221, hsa-miR-222, act as hubs in the constructed networks. We also confirm the biological relevance of the constructed networks through literature review and functional analysis.

## Results

### Data and experimental settings

In this study, miRNA and mRNA expression profiles obtained from the MSKCC Prostate Oncogenome Project [36] were matched at three stages of prostate cancer. The dataset contains 373 miRNAs and 19,780 mRNAs from 27 normal, 98 primary and 13 metastatic stages. During preprocessing, sample-wise and feature-wise normalization was conducted, and miRNAs and mRNAs were separately normalized. The experimental parameter settings are listed in Table 1. The parameters are those yielding optimal performance in empirical experiments. A hypergraph can include hyperedges with different number of genetic variables but we fixed the number of variables for all hyperedges of a hypergraph in this study.

**Table 1:** Parameter settings for experiments

**Parameters**	**Values**	**Parameters**	**Values**
# of miRNA	**3**	# of mRNA	**5**
# of modules	**variable**	*β* in (5)	**1.0**
Epochs of structure learning	**100**	Epochs of parameter learning	**20**
*η* in (10)	**1.0**	*κ* in (11)	**1.0**
γ in (13)	**1.0**	*R*_max_ , *R*_min_	**0.9, 0.5**

### Classification performance

**Figure 2:**
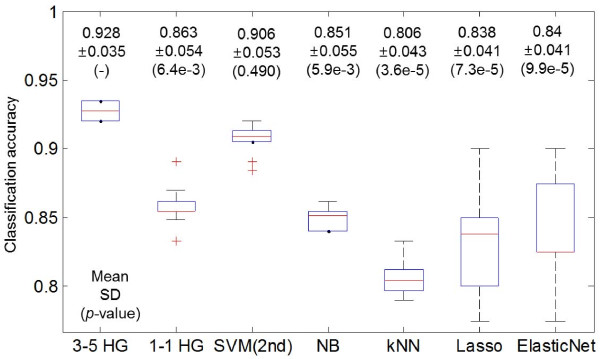
**Boxplots of classification accuracy on the test set.***m-n* HG denotes the hypergraph-based model whose all hyperedges embody *m* miRNAs and *n* mRNAs. All results are averaged after 10 runs by 10-fold cross validation. *P*-values are calculated using *t*-test of our model and other models.

Classification performance was evaluated using three standard classification models; support vector machines (SVMs) with the 2nd polynomial kernel and sequential minimal optimization (SMO), *k*-th nearest neighbor classifiers (*k*-NNs), and naïve Bayes classifiers (NBs) implemented in Weka [37]. The MATLB algorithms lasso and elastic net (*α*=0.5) were also used. All results were averaged over 10 experiments. Figure 2 presents the classification accuracy of our model compared to other models. As revealed by the *p*-values of the *t*-test, the proposed hypergraph-based model competes on-par with SVMs and outperforms the *k*-NN, NB and Lasso-based methods. In addition, by comparing the results of 3–5 HG (a hypergraph model whose hyperedges consist of three miRNAs and five mRNAs) and 1–1 HG, we observe that higher-order relationships are more important for discriminating cancer stages than pair-wise relationships between a single miRNA and mRNA.

### Model evaluation

The proposed hypergraph-based learning method is evaluated on simulation data for verifying whether the method finds true solutions. The data consist of 500 instances with 7 variables whose mean is zero and the class label of each instance is determined as follows:

$$x_{i}∼N\left(0,1\right),\;1\leq i\leq 7$$

(1) $$c^{\left(n\right)}=\left\{\begin{array}{c} 1,\;\mathrm{if}\;x_{2}>2\wedge x_{3}>2\wedge x_{4}>2\\ \;2,\;\mathrm{if}\;x_{5}<-2\wedge x_{6}<-2\wedge x_{7}<-2\\ \;3,\;\mathrm{otherwise} \end{array}\right)\;,$$

where *x*_*i*_ and *c*^(*n*)^ denote the *i*-th random variable and the class label of the *n*-th instance. Table 2 illustrates the classification accuracy and predefined modules in the learned model. The accuracy is averaged after 10 experiments by 10-fold cross validation, and each hypergraph includes 20 hyperedges with four variables. In Table 2, Module 1 and 2 means the number of case when there exist hyperedges involving a predefined-set 1 (*x*_*2*_, *x*_*3*_, *x*_*4*_) and 2 (*x*_*5*_, *x*_*6*_, *x*_*7*_) in a learned hypergraph. Because we conducted 10-fold cross validation, the maximum values of Module 1 and 2 are ten. Therefore, we indicate that our method can find true solutions from small combinatorial spaces, considering the accuracy and the number of found variable modules.

**Table 2:** Verification result on the simulation dataset

**Models**	**SVM**	**DT**	**kNN**	**HG**	**Module 1**	**Module 2**
Accuracy	**0.956**	**0.886**	**0.93**	**0.956**	**10**	**10**
±SD	**±0.002**	**±0.004**	**±0.006**	**±0.003**	**-**	**-**

**Figure 3:**
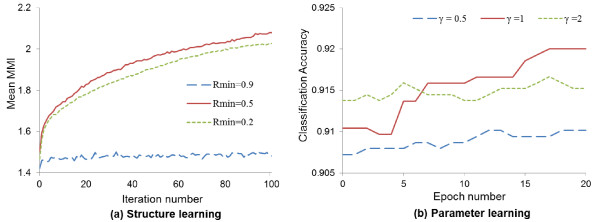
**Learning curves in the structure and the parameter learning phases.** As the performance measure, we used mean multivariate mutual information (MMI) of all hyperedges in the model for the structure learning and accuracy on 10 fold cross validation for the parameter learning. *Rmax* is fixed as 0.9 in **(a)** and *γ* is a learning rate for the parameter learning in **(b)**. All results are averaged on 10 experiments of 10- fold cross validation.

Figure 3 presents two learning curves under various conditions of the structure (a) and the parameter (b) learning phases. As the measure for structure learning, we used mean multivariate mutual information (MMI) of all hyperedges in the model because the goal of the structure learning is to find the significant higher-order cancer-specific gene interaction modules, and an MMI is the measure reflecting the strength of interactions among genetic factors in the hyperedges considering the stage of cancer. On the other hand, classification accuracy is used as the measure for the parameter learning phase since the weight for each cancer stage is updated to minimize the error in the phase. Figure 3(a) presents the increase of mean MMI under various *Rmin* which is the minimum ratio of the hyperedges replaced in the iteration, and plays a role of the structure learning rate. We indicate that too large an *Rmin* causes low MMI by replacing too many hyperedges and too small an *Rmin* leads slow increase of the MMI from Figure 3(a). Figure 3(b) presents similar results to (a) with respect to the effect of learning rate γ.

**Figure 4:**
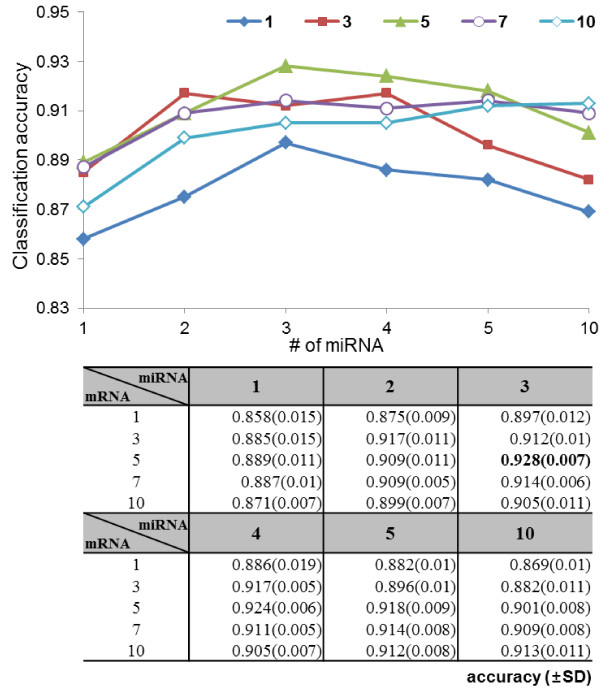
**Classification accuracy according to the number of miRNA and mRNA in the hyperedges.** The classification accuracy is the best when a hypergraph consists of hyperedges with three miRNAs and five mRNAs. All results are averaged on 10 experiments of 10- fold cross validation.

Moreover, Figure 4 shows the classification accuracy according to the number of genetic factors in the hyperedges. The classification accuracy is the best when a hypergraph consists of hyperedges with three miRNAs and five mRNAs. We indicate that small number of genetic variables show worse performance because various processes of prostate cancer is influenced on the complex interactions among many features. Furthermore, the accuracy of the hypergraphs including hyperedges with more than ten genetic variables is low since the models consist of too specific information and thus have the low generalization property.

**Figure 5:**
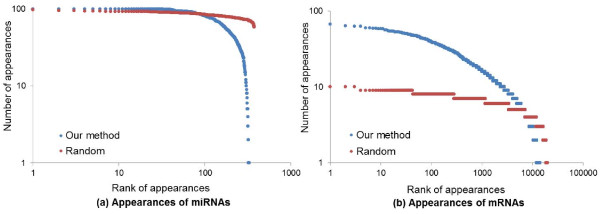
**Reproducibility of decisive miRNAs (a) and mRNAs (b) influencing on classification.** 100 hypergraphs are generated by randomly selecting miRNAs and genes, while another 100 hypergraphs are generated by our learning method (10 experiments with 10-fold cross validation). Each hypergraph includes 200 hyperedges consisting of three miRNAs and five mRNAs. The x-axis denotes the rank of the appearance of miRNAs or mRNAs, and y-axis is the number of miRNA or mRNA appearances. Both axes are log-scaled.

Table 3 and Figure 5 show that the proposed learning method can stably extract significant genetic factors despite its random selection approach. We define a measure as the number of appearance of a gene in the model, *A*(*x*_i_), for verifying the stability of the model as follows:

$$A\left(x_{i}\right)=\sum_{m=1}^{100}\delta \left(x_{i},H_{m}\right),$$

(2) $$\delta \left(x_{i},H_{m}\right)=\left\{\begin{array}{c} 0\;\mathrm{if}\;x_{i}\;\mathrm{is}\;\mathrm{not}\;\mathrm{involved}\;\mathrm{in}\;H_{m}\\ 1\;\mathrm{otherwise} \end{array}\right)\;,$$

where *x*_*i*_ denotes the *i*-th miRNA or mRNA, and *H*_*m*_ is the *m*-th learned model. *δ*(*x*_*i*_, *H*_*m*_) is an indicator function and it returns one when *x*_*i*_ appears at least once in *H*_*m*_, otherwise zero. The proposed method is compared to randomly generated hypergraphs each comprising 200 hyperedges involving three miRNAs and five mRNAs. The results are derived from 100 models learned by 10 experiments of 10-fold cross validations, and 100 randomly generated hypergraphs. According to Figure 5(a), our method extracts significant miRNAs only, while almost all of the miRNAs are involved in random graphs. Moreover, whereas the learning method selects several significant mRNAs, all mRNAs appear at low frequency in the random graphs, as shown to Figure 5(b). The stability and reproducibility of the proposed model is evident from the high-frequency occurrence of high ranked miRNAs and mRNAs, indicating that certain genes persist in the models. Table 3 lists the miRNAs and mRNAs that appear frequently and rarely in 100 learned models and in randomly generated graphs. Given that several key genes decisively affect a specific cancer, we posit that the proposed model consistently selects essential factors, in contrast to a random selection.

**Table 3:** Frequently and rarely appearing miRNAs and mRNAs in the 100 learned models

**Our method**		**Random**		**Our method**		**Random**	
Frequent	# of	Frequent	# of	Rare	# of	Rare	# of
miRNAs	appearances	miRNAs	appearances	miRNAs	appearances	miRNAs	appearances
miR-1	100/100	miR-152	97/100	miR-95	0/100	miR-30a	58/100
miR-100	100/100	miR-1	95/100	miR-937	0/100	miR-134	60/100
miR-133a	100/100	miR-486-5p	95/100	miR-933	0/100	miR-106a	60/100
miR-143	100/100	miR-199b-5p	94/100	miR-887	0/100	miR-362-5p	63/100
miR-145	100/100	miR-377	94/100	miR-744	0/100	miR-200b	63/100
**Our method**				**Random**			
Frequent	# of	Frequent	# of	Frequent	# of	Frequent	# of
mRNAs	appearances	mRNAs	appearances	mRNAs	appearances	mRNAs	appearances
ACTA2	67/100	ILK	60/100	AIPL1	10/100	CACNA1D	9/100
SVIL	64/100	CSRP1	59/100	CBY3	10/100	CDC25C	9/100
ACTN1	63/100	TPM1	59/100	SHKBP1	10/100	DHRS7C	9/100
CAV1	63/100	FRMD6	58/100	ADCY5	9/100	FAT3	9/100
CCND2	60/100	LOC645954	58/100	C17orf58	9/100	FOXN3	9/100

### Constructed higher-order miRNA-mRNA interaction networks in prostate cancer

**Figure 6:**
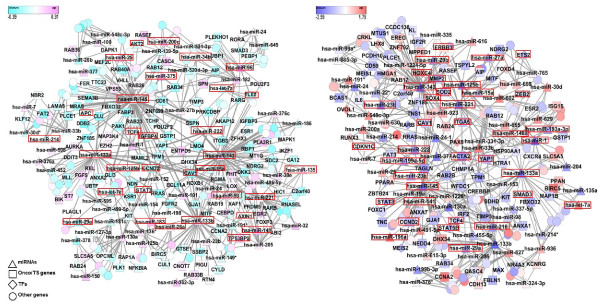
**Constructed (a) primary prostate cancer-specific and (b) metastatic prostate cancer-specific miRNA-mRNA interaction networks.** The primary-specific network includes 67 miRNAs and 233 mRNAs, while the metastatic network involves 65 miRNAs and 180 mRNAs. Both networks include 500 bi-relational edges which are selected based on their summed weight (among all edges converted from 20000 hyperedges of 100 hypergraphs). Up- and down-expressed miRNAs and genes are determined by the mean of each stage class. The red boxed miRNAs and genes have been reported to be associated with the particular stage of prostate cancer. The triangles, rectangles, diamonds and circles denote miRNAs, oncogenes or tumor suppressor genes, transcription factors, and other genes in the network, respectively.

The miRNA-mRNA interaction network constructed from the proposed model is illustrated in Figure 6(a) and (b) for primary and metastatic prostate cancer respectively [38]. The constructed interaction networks comprise putative miRNA-mRNA modules associated with each stage of prostate cancer, and reflect their higher-order relationships. The primary prostate cancer network includes 67 miRNAs and 233 mRNAs, while the metastatic prostate cancer network involves 65 miRNAs and 180 mRNAs.

**Figure 7:**
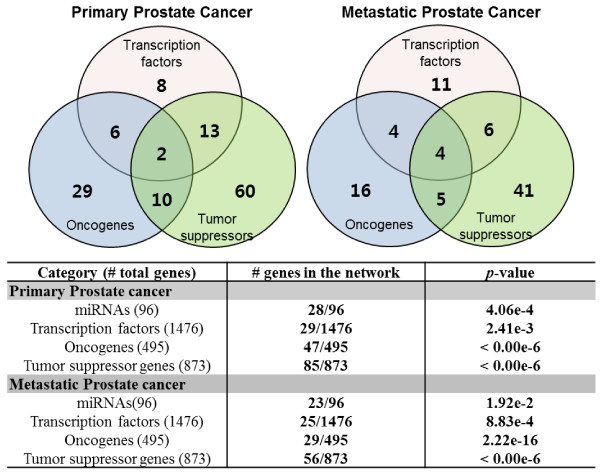
**The miRNAs and mRNAs in the constructed networks are enriched in cancer-related genes with a significant *p*-value.**

Many of the miRNAs in the constructed networks have been significantly associated with prostate cancer in the literature, and are thus termed prostate cancer-related miRNAs [39]. In addition, many of the genes in the constructed networks overlap with cancer-related genes, including transcription factors. To confirm this finding, we compiled a list of 496 oncogenes and 874 tumor suppressor genes from the Cancer Genes of Memorial Sloan-Kettering Cancer Center [40] and 1476 human transcription factors [41]. We investigated cancer gene enrichment in the constructed interaction networks by hypergeometric test. As shown in Figure 7, most of the significant genes (*p*-value close to 0) in the constructed networks are overrepresented in the compiled list. This result unambiguously demonstrates that our model can build interaction networks of genetic factors associated with cancer processes.

Interestingly, the enriched hyperedges, and the expression levels of the miRNAs and mRNAs, differ considerably between the primary and metastatic networks. Up- and down-expressed miRNAs and genes are determined by their means at each stage. The red boxed miRNAs and genes are known to be associated with the various stages of prostate cancer [4-8,42,43]. The triangles rectangles, diamonds and circles denote miRNAs, oncogenes/ tumor suppressor genes, transcription factors, and other genes in the network, respectively.

### Functional analysis of the constructed interaction networks

The constructed miRNA-mRNA interaction networks were validated by functional analyses based on a literature review and gene set analysis. As mentioned above, many of the miRNAs and mRNAs involved in the identified interactions are known indicators of prostate cancer [4-8]. In addition, the mRNAs comprise a portion of their predicted target genes [44], some of which have been experimentally validated. In particular, several miRNAs are known as ‘oncomiRs’ which function as oncogenes or tumor suppressors, including has-miR-1, -133a, -143, -145, -221, and −222 [45-48]. Many hyperedges in the constructed networks contain the above miRNAs as their components; these particular miRNAs also act as hubs in the networks.

Especially, hsa-miR-143 and hsa-miR-145 play a crucial role in metastatic prostate cancer, and are recognized as a clinicopathological signature of prostate cancer [47]. Interaction modules involving hsa-miR-143 and −145 occupy a large portion of the networks constructed by our model. In addtion, the identified interactions in metastatic prostate cancer contain several experimentally confirmed targets of hsa-miR-143 and −145, including CLINT1, CDKN1A, IRS1, MAPK7, PPM1D and SOD2. Furthermore, hsa-miR-143 and −145 are expressed at low levels in the metastatic network, as has been experimentally validated [7].

Moreover, hsa-miR-200c emerges as a distinct miRNA in the network of primary prostate cancer. According to several studies, hsa-miR-200c overexpression inhibits metastasis prostate cancer, while aberrant regulation triggers the invasion and migration of prostate cancer at the post-transcriptional level [49].

Our model identified several transcription factors associated with prostate cancer metastasis, such as ETS2, HOXC4, STAT3, STAT5B, SOX4 and ZEB2. Among these, SOX4, STAT3 and STAT5B are known regulators of metastatic prostate cancer through the regulation of genes involved in miRNA processing, transcriptional regulation, and developmental pathways [50-52]. Indeed, SOX4 is directly regulated by hsa-miR-335 in cancer progression [50], while hsa-miR-125b coordinates STAT3 regulation in the proliferation of tumor cells [51,53].

Interactions involving hsa-miR-29b/MMP2 and hsa-miR-335/SOX4 appear concurrently in the constructed metastatic network (Table 4). This finding is consistent with previous studies, in which-miR-29b and −335 were found to suppress tumor metastasis and migration by regulating MMP2 and SOX4, respectively [42,54]. Interestingly, both of these interactions involve hsa-miR-143, which is closely linked to prostate cancer progression. Furthermore, the well-known cancer-associated genetic factors MMP2 and SOX4 co-emerged in the identified interactions. Although the interactions identified by our model have not been previously reported, they clearly reflect higher-order relationships between miRNAs and mRNAs. As such, they may signify unknown regulatory circuits in prostate cancer development and progression. This result suggests the utility of the proposed model in identifying undiscovered miRNA-mRNA interactions.

**Table 4:** Examples of modules (hyperedges) in primary and metastatic prostate cancer

**miRNAs [exp. levels: up (+), down (−)]**			**mRNAs [exp. levels: up (+), down (−)]**				
**Primary prostate cancer**							
hsa-miR-330-3p(−)	**hsa-miR-133b**(+)	**hsa-miR-222**(−)	**MAP1B**(−)	**WWC3**(−)	CAV1(−)	DHX35(−)	TSHZ3(−)
hsa-miR-143(+)	hsa-miR-502-5p(−)	**hsa-miR-548c-3p**(+)	**ZZEF1**(−)	**C20orf194**(−)	**TSPYL2**(−)	MBD3(+)	GPR132(+)
**hsa-miR-19a**(+)	**hsa-miR-133a**(+)	hsa-miR-153(+)	**BMPR1B**(+)	**WWC3**(−)	PCBP4(−)	TCEAL4(−)	CUL4A(+)
**hsa-miR-130a**(+)	**hsa-miR-375**(+)	**hsa-miR-19a**(+)	**RAP1A**(−)	CYLD(−)	SNORA71D(+)	NDUFA6(−)	RGS9BP(−)
**hsa-miR-221**(−)	**hsa-miR-106b**(+)	**hsa-miR-222**(−)	**ARSJ**(−)	SSPN(−)	C3orf58(+)	PTGDS(−)	RARB(−)
**hsa-miR-130a**(+)	**hsa-miR-133a**(+)	**hsa-miR-19a**(+)	**VNN1**(−)	FGF5(+)	ELOVL7(+)	PHPT1(−)	RND3(−)
**hsa-miR-133a**(+)	**hsa-miR-222**(−)	**hsa-miR-130a**(+)	**C10orf137**(+)	FAM108C1(+)	SCRIB(+)	PRKAR1A(−)	MOXD1(−)
**hsa-miR-130a**(+)	hsa-miR-149*(−)	**hsa-miR-26a**(+)	**RASEF**(+)	TPM1(−)	CRB2(−)	TMEM132A(+)	LIX1L(−)
**hsa-miR-133b**(+)	**hsa-miR-23b**(+)	**hsa-miR-106b**(+)	**PFAS**(+)	UNC5C(−)	HLF(−)	PSEN1(+)	EZH2(+)
hsa-miR-145(+)	hsa-miR-200c(+)	hsa-miR-23b(+)	TTC23(−)	PARM1 (−)	TOPORS(+)	NEBL(−)	RCAN2(−)
**Metastatic prostate cancer**							
**hsa-miR-221**(−)	**hsa-miR-29b**(−)	**hsa-miR-143**(−)	**SOX4**(+)	**MMP2**(−)	**RASEF**(−)	**SOD2**(−)	SCN9A(+)
hsa-miR-29b(−)	**hsa-miR-335**(−)	**hsa-miR-143**(−)	**SOX4**(+)	**MPPED1**(+)	**ERBB3**(+)	HOXC4(+)	SMTN(−)
hsa-miR-143(−)	hsa-miR-22*(−)	hsa-miR-23b(−)	**CDKN1A**(−)	**HMGA1**(+)	**PELO**(−)	**RAB17**(+)	TMEM150(+)
**hsa-miR-125b**(−)	hsa-miR-616(+)	**hsa-miR-143**(−)	**TSPYL2**(−)	**ERBB3**(+)	**ACAD8**(−)	**PHF15**(+)	TMEM16G(−)
hsa-miR-19a(−)	**hsa-miR-141**(+)	**hsa-miR-145**(−)	**PCDH20**(+)	**DNAJC3**(−)	STAT3(−)	ZNF385(+)	ACTA2(−)
hsa-miR-133b(−)	**hsa-miR-145**(−)	**hsa-miR-218**(−)	**IRF2**(−)	**TCF4**(−)	**STAT5B**(−)	RAB2B(−)	WFDC1(−)
**hsa-miR-143**(−)	**hsa-miR-145**(−)	hsa-miR-222(−)	**ITGA5**(−)	**MAPK7**(+)	MAP3K2(−)	RAB34(−)	S100A1(+)
**hsa-miR-143**(−)	**hsa-miR-145**(−)	**hsa-miR-214**(−)	**FEM1A**(+)	**ITGA5**(−)	**NAGPA**(+)	C1orf142(+)	ERAS(+)
**hsa-miR-143**(−)	**hsa-miR-193b**(−)	**hsa-miR-145**(−)	**CLINT1**(−)	**GJA1**(−)	**MAPK7**(+)	RARRES2(−)	IL28A(+)
hsa-miR-221(−)	**hsa-miR-1**(−)	hsa-miR-133b(−)	**TPM1**(−)	**NDFIP2**(−)	**RAD17**(−)	**VPS28**(+)	**INPPd5E**(+)

miRNAs and their predicted targets are given in bold font. The underlined genes are the cancer genes archived in the Memorial Sloan-Kettering Cancer Center.

To confirm the biological relevance of the constructed interaction networks, we analyzed the functional correlations among the network genes by canonical pathway analysis [55]. The significant (low *p*-value) results of the analysis for the primary and metastatic prostate cancer networks are summarized in Table 5. Many of the enriched pathways are closely associated with prostate tumorigenesis and metastasis. In particular, the *β*-catenin degradation pathway, the Wnt/*β*-catenin pathway and the Wnt canonical pathway are associated with Wnt signaling, which regulates many genes implicated in prostate cancer. These pathways were identified as significant in the primary prostate cancer network. Deregulation of the Wnt-related pathway reportedly affects prostate cell proliferation and differentiation [56]. Moreover, the annotated genes in the constructed network, such as APC, AXIN1, AKT2, CCND2, CAV1, TLE2 and TCF4, are essential regulatory components of these pathways in prostate cancer. ErbB-related pathways were identified in the metastatic network, including the ErbB network pathway, ErbB4 pathway, Her2 pathway, ErbB2/ErbB3 signaling pathway and the EGFR pathway, which are implicated in prostate cancer progression and metastasis [43,57]. The FOXM1 pathway also regulates tumor metastasis (including that of prostate cancer) by stimulating the expression of several genes involved in the proliferation of tumor cells and cell cycle progression [58]. The top-ranked pathway in the metastatic network is the MYC activation pathway. MYC reportedly promotes the metastatic phenotype by altering the epigenetic landscape of cancer cells, and is overexpressed in ~75% of advanced prostate cancer patients [43]. Thus, the MYC pathway is a putative key feature of metastatic progression [59].

**Table 5:** Canonical pathway analysis of the constructed interaction networks in primary and metastatic prostate cancer

**Canonical pathway analysis**	***p*-value (<0.05)**
**Primary prostate cancer**	
Pathways in cancer	1.70e-03
Rb1 pathway	5.95e-03
Retinoic acid pathway	6.61e-03
Aurora A pathway	7.44e-03
Beta-catenin degradation pathway	9.95e-03
Wnt/beta-catenin pathway	1.03e-02
Wnt canonical signaling pathway	1.34e-02
Met pathway (signaling of HGF receptor)	1.39e-02
P38-alpha/beta downstream pathway	1.52e-02
Beta-catenin nuclear pathway	1.58e-02
Aurora B pathway	1.66e-02
EPHB forward pathway	1.81e-02
IFN-gamma pathway	1.81e-02
P53 hypoxia pathway	1.97e-02
MYC repress pathway	2.15e-02
Progesterone mediated oocyte maturation	2.19e-02
Rac CycD pathway (Ras and Rho protein on G1/S transition)	2.73e-02
PLK1 pathway	2.88e-02
IL-6 (interleukin-6) pathway	3.08e-02
FGFR2C ligand binding and activation	3.58e-02
Cell cycle	4.43e-02
PDGFR-beta signaling pathway	4.59e-02
**Metastatic prostate cancer**	
MYC activate pathway	1.41e-04
ErbB network pathway	2.78e-03
KIT receptor signaling pathway	3.28e-03
IL-10 pathway	4.40e-03
Pathways in cancer	4.76e-03
ErbB4 pathway	6.12e-03
Her2 pathway (ErbB2 in signal transduction and oncology)	8.51e-03
Yap1 and Wwtr1/Taz stimulated gene expression	1.09e-02
Smooth Muscle Contraction	1.22e-02
Barrestin pathway	1.53e-02
IL-6 signaling pathway	1.85e-02
STAT3 pathway	1.85e-02
IL-2/STAT5 pathway	2.00e-02
RAS pathway	2.00e-02
ErbB2/ErbB3 signaling pathway	2.19e-02
Syndecan4 pathway	2.38e-02
PPAR-alpha pathway	2.61e-02
Integrin signaling pathway	3.72e-02
Rela pathway	3.78e-02
HDAC class I pathway	3.94e-02
FOXM1 pathway	4.24e-02
IL-7 pathway	4.23e-02
EGFR pathway	4.70e-02

## Discussion

The proposed hypergraph-based model characterizes higher-order interactions among heterogeneous genetic factors from archived data. Human cancers are typically caused by the modular control of multiple genetic factors. By analyzing gene relationships at higher-order levels, thus, we can better understand the behavior of complex cancer mechanisms. Moreover, the cooperative activities and the combinatorial regulations governed by miRNAs and mRNAs are largely unknown. We have demonstrated that higher-order relationships discriminate between specific cancer stages more precisely than pair-wise analyzes of single miRNA and mRNA interactions. From this viewpoint, we can construct a more complete interaction network consisting of putative biologically significant miRNA-mRNA modules.

In addition, our method focuses on discovering potential interactions in unknown miRNA-mRNA regulatory circuits related to specific cancer stages without the known biological information [60,61]. The proposed model finds statistically significant gene modules from given expression profiles using a data-driven approach with co-regulatory measure (mutual information). However, a similar hypergraph structure could be readily constructed from other types of quantitative biological information, such as miRNA-target information and gene sequence similarity values. Furthermore, the hypergraph-based model more flexibly represents miRNA-RNA interactions than other methods (which assume that the expression states of miRNAs and mRNAs are linearly proportional to each other), because it isolates significant modules from the statistical co-expressed pattern among genes at a higher-order level.

The proposed hypergraph-based model is similar to Bonnet’s *et al.*[26,27] and Li *et al.*[28], where higher-order relationships governed by miRNA-mRNA interactions are inferred solely from expression profiles. Bonnet’s method is based on a clustering approach, it cannot readily infer gene regulatory modules at a specific cancer stage. In contrast to Bonnet’s method, our method explicitly considers the sample status, (the primary or metastatic state of prostate cancer), from which it constructs cancer stage-specific networks. Liu’s approach is based on Corr-LDA, which requires that data are discretized. By contrast, our method uses intact real-valued data, thus preventing the information loss caused by the discretization.

Furthermore, the proposed model finds the true solution in a small subset of the features, because the problem space is small enough to search exhaustively. Also, unlike other models, our model can efficiently handle the very high-dimensional data required for complex higher-order interactions among features. However, the limitation of the proposed hypergraph-based model emerges at small sample sizes. If the data are few, the reliability of the mean and covariance defined in a hyperedge is reduced.

## Conclusions

We have proposed a hypergraph-based model consisting of higher-order miRNA-mRNA modules, which allows the construction of biologically meaningful interaction networks associated with specific cancer stages. For identifying potential significant interactions and refining model performance, we introduced a two-phase learning approach comprising structure and parameter learning. Finally, we constructed cancer stage-specific interaction networks reflecting higher-order miRNA and mRNA relationships by converting the hypergraph structure into an ordinary graph.

We constructed higher-order miRNA-mRNA interaction networks associated with the specific stage of prostate cancer from a matched dataset using the proposed model. The performance of the proposed model is similar to that of SVMs and superior to other classification models (outperforming them by approximately 6–10%). More importantly, our model can construct carcinogenic miRNA-hubbed networks that characterize primary and metastatic prostate cancer. Furthermore, we demonstrated that a large proportion of the miRNAs and mRNAs identified in the constructed interaction networks are indeed involved in prostate cancer progression and development. The proposed hypergraph-based model therefore presents as an alternative method for discovering potential gene regulatory circuits. Such discoveries will greatly assist our understanding of cancer pathogenesis.

## Methods

### Hypergraph-based models

A hypergraph-based model characterizes complex interactions among many genetic factors using hypergraph structures. A hypergraph generalizes the edge concept to a hyperedge by which more than two variables can be connected simultaneously [62,63]. As such, it is suitable for representing higher-order relationships among heterogeneous features (e.g. miRNAs and mRNAs). In our model, a hyperedge contains two or more variables corresponding to miRNAs and mRNAs, weighted by the strength of the higher-order dependency among its elements for each class (where the class denotes a specific cancer stage). Thus, each hyperedge implies a set of miRNA-mRNA modules associated with a certain stage of cancer. The proposed model therefore facilitates the construction of higher-order miRNA-mRNA interaction networks among a population of candidate gene modules related to a specific cancer stage.

**Figure 8:**
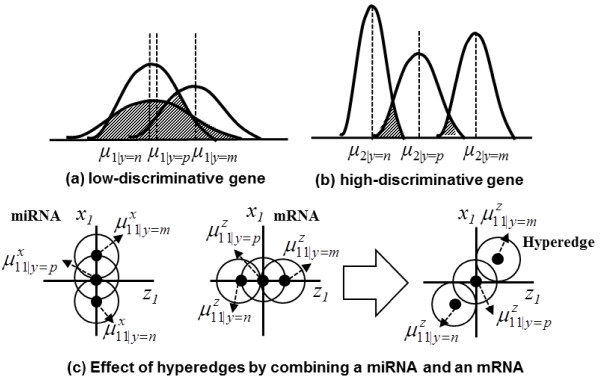
**Biological meaning of mean and variance used in representing a hyperedge.** Panels **(a)** and **(b)** illustrate how the means and variances differ between low and high discriminative genetic factors. A gene is low-discriminative when the means are similar at each disease stage but the variances are large (where *n*, *p*, and *m* denote normal, primary, and metastatic stage, respectively). Panel **(c)** illustrates the enhanced discriminative capability of a hyperedge involving two genetic factors. By comparing the discriminative capability of each miRNA or mRNA, the discrimination capability of the hyperedge is enhanced.

A hypergraph-based model *H* is formally defined as a triple *H* = (*X*, *Z*, *E*) where *X*, *Z*, and *E* denote the sets of miRNAs, mRNAs, and hyperedges, respectively. A hyperedge is represented by a set of statistical values, including mean and covariance for the class label corresponding to a cancer stage. The mean gene expression values differ widely among the class labels, implying that gene expression depends on cancer progression, as shown in Figure 8. The hyperedge approach enhances the discriminative capability by combining miRNAs and mRNAs (Figure 8). Given an expression dataset with *N* instances $D={\left\{d^{\left(n\right)}\right\}}_{n=1}^{N}={\left\{x^{\left(n\right)},z^{\left(n\right)},y^{\left(n\right)}\right\}}_{n=1}^{N}$, where **x**^(*n*)^ and **z**^(*n*)^ are real-valued vectors of miRNA and mRNA expressions in the *n*-th instance, and *y* is an element of a cancer stage set *Y*, the *i*-th hyperedge *e*_*i*_ contains the mean vectors and the covariance of its miRNAs and mRNAs for the given cancer stage:

(3) $$e_{i}=\left\{\begin{array}{l} e_{i|y=y_{1}}\\ \;\ldots \\ e_{i|y=y_{\left|Y\right|}} \end{array}\right\}=\left\{\begin{array}{l} {\left(\mu_{i},\Sigma_{i}\right)}_{|y=y_{1}}\\ \;\ldots \\ {\left(\mu_{i},\Sigma_{i}\right)}_{|y=y_{\left|Y\right|}} \end{array}\right\},$$

(4) $$\mu_{i}=\left(\mu_{i1}^{x},\ldots ,\mu_{\mathrm{il}}^{x},\mu_{i1}^{z},\ldots ,\mu_{\mathrm{im}}^{z}\right)\;\mathrm{and}\;l+m=|e_{i}|$$

where $\mu_{\mathrm{ij}}^{x}$ and $\mu_{\mathrm{ik}}^{z}$ denote the means calculated from the expression profiles of the *j*-th miRNA and the *k*-th mRNA, respectively, in the *i*-th hyperedge (whose elements comprise *l* miRNA and *m* mRNAs). *l* and *m* are called the degrees of miRNA and mRNA of the hyperedge, respectively. By the definition of a hyperedge, each hyperedge has |*Y*| mean vector /covariance pairs, and |*Y*| weights. The hypergraph-based model is considered as a population of hyperedges. Given a gene expression profile **(x**, **z**), the cancer stage of the profile is classified as y*, for which the summation of the expected values (the products of the hyperedge weight and the probability of **(x**, **z**) matching the hyperedge), is highest among the elements of *Y*. “**(x**, **z**) matches *e*_*i|y*_” means that **(x**, **z**) has similar expression values to ones of the *i*-th hyperedge with respect to the genetic variables involved in *e*_*i|y*_ at cancer stage *y*, and we introduce a Gaussian kernel into the hyperedge to calculate the matching probability of **(x**, **z**) and *e*_*i|y*_, *P*(*u*=1|**x**, **z**, *e*_*i|y*_). The matching probability is calculated by the normalized subdimensional distance between *e*_*i|y*_ and **(x**, **z**):

(5) $$P\left(u=1|x,z,e_{i|y}\right)=\exp\left\{-\mathrm{\beta d}\left(x,z,e_{i|y}\right)\right\},$$

(6) $$d\left(x,z,e_{i|y}\right)=\frac{1}{\left|e_{i}\right|}{\left\{\sum_{j=1}^{l}\frac{{\left(x_{\mathrm{ij}}-\mu_{\mathrm{ij}}^{x}\right)}^{2}}{{\left(\sigma_{\mathrm{ij}|y}^{x}\right)}^{2}}+\sum_{k=1}^{m}\frac{{\left(z_{\mathrm{ik}}-\mu_{\mathrm{ik}}^{z}\right)}^{2}}{{\left(\sigma_{\mathrm{ik}|y}^{z}\right)}^{2}}\right\}}^{\frac{1}{2}},$$

where *u*=1 denotes that **(x**, **z**) matches *e*_*i|y*_ , $\sigma_{\mathrm{ij}|y}^{x}$ and $\sigma_{\mathrm{ij}|y}^{z}$ are the standard deviations of **x**_*ij*_ and **z**_*ik*_ (the *j*-th miRNA and *k*-th mRNA, respectively) in the *i*-th hyperedge for a given *y*, and *β* is a constant for adjusting the probability. Larger *β* implies smaller matching probability, and therefore a smaller number of hyperedges influence on classifying the data. Specifically, the cancer stage y* of **(x**, **z**) is computed as follows:

1. Calculate *c*_*y* '_, the sum of the expected values for each *y* ' in *Y* over all hyperedges of *H*:

(7) $$c_{y'}=\sum_{i=1}^{\left|H\right|}w\left(e_{i|y=y'}\right)P\left(u=1|x,z,e_{i|y=y'}\right),$$

where |*H*| denotes the number of hyperedges and *w*(*e*_*i|y*_) is the weight of *e*_*i|y*_, explained in the next subsection.

2. Predict the cancer stage as y*:

(8) $$y^{*}=\underset{\;y'\in Y}{\arg\;\max}c_{y'}.$$

In terms of distance-based connectionist models, our model is related to radial basis function networks (RBFNs) [64]. Whereas RBFNs use kernelized distance for all variables, the proposed hypergraph model uses the probability derived from the subdimensional distance on the projected space corresponding to each hyperedge. Unlike RBFNs, therefore, the hypergraph-based model can detect embedded subpatterns reflecting higher-order relationships among the components. Because these embedded subpatterns influence the classification, we can intuitively analyze the complex interactions of genetic factors that contribute to classifying a specific cancer stage.

### Learning hypergraph-based models

The proposed model learns by finding a hypergraph structure with high discriminative capability at a specific cancer stage. This is achieved by maximizing the conditional likelihood for a model *H* and the gene expression profiles and a log function is adopted for convenience. To minimize the error of classifying the cancer stage, *E*_*D*,*H*_ , the log conditional likelihood is maximized by least mean square criteria using (7) and a sigmoidal function:

(9) $$\begin{array}{l} H*={\underset{H}{\arg\;\max}}_{\log\prod_{n=1}^{N}p\left(y^{\left(n\right)}|x^{\left(n\right)},z^{\left(n\right)},H\right)}\\ \;=\underset{H}{\arg\;\max}\sum_{n=1}^{N}\log p\left(y^{\left(n\right)}|x^{\left(n\right)},z^{\left(n\right)},H\right)\\ \;\equiv \underset{H}{\arg\;\max}\sum_{n=1}^{N}\delta \left(y^{\left(n\right)},y_{H}^{'}\right)\\ \;=\underset{H}{\arg\;\min}E_{D,H}\;\;\approx \underset{H}{\arg\;\min}\sum_{n=1}^{N}\sum_{y'\in Y}\\ \;\times {\left(\delta \left(y^{\left(n\right)},y'\right)-P\left(y'|x^{\left(n\right)},z^{\left(n\right)},H\right)\right)}^{2} \end{array}$$

s.t.

$$P\left(y'|x,z,H\right)={\left\{1+\exp\left(c_{y'}-\frac{1}{\left|Y\right|}\cdot \sum_{y\in Y}c_{y}\right)\right\}}^{-1}\;$$

where (*x*^(*n*)^, *z*^(*n*)^) denotes the *n*-th miRNA-mRNA expression and *y*^(*n*)^ is the cancer stage of the example. $y_{H}^{'}$ is the label predicted by *H* and *δ*(*y*(*n*), $y_{H}^{'}$) is an indicator function, equal to 1 if *y*(*n*) equals $y_{H}^{'}$, and 0 otherwise. To enhance the classification accuracy, it is essential that the population comprises hyperedges with high discriminative capability, and the hyperedge weights must be refined to minimize (9) in the generated hypergraph.

To meet these requirements, the learning iterates two phases: structure learning and parameter learning. The structure learning constructs a hypergraph from hyperedges that identify potential miRNA-mRNA modules. The weights of the hyperedges are updated to minimize the classification error of the generated gene module population during the parameter learning phase. Because the hypergraph-based model represents a huge combinatorial feature space (size 2^|x|+|z|^) of many miRNAs and mRNAs, exhaustively searching for the optimal population is infeasible. Instead we adopt an evolutionary learning method based on information-theoretic criteria to generate putative hyperedges for the structure learning.

We assume that a hyperedge consisting of strongly interactive miRNAs and mRNAs is highly discriminative for classification in this study. Mutual information is used as a co-regulatory measuring criterion for efficiently selecting genes for hyperedge generation. Mutual information (MI) is an information-theoretic measure that specifies the degree of conditional independency between two random variables. When a genetic factor more strongly determines the cancer stage, the MI between the gene and the cancer stage is increased. A hyperedge is generated by probabilistically selecting miRNAs and mRNAs, and the MI between each gene and the class label determines the probability of selecting the genes. The probability *P*_*I*_(*X*_*i*_) of selecting the *i*-th gene *X*_*i*_ is defined such that miRNAs or mRNAs with high MI are selected more frequently:

(10) $$P_{I}\left(X_{i}\right)=\frac{{\left\{I\left(X_{i};Y\right)\right\}}^{\eta }}{\sum_{X_{i}\in X}{\left\{I\left(X_{i};Y\right)\right\}}^{\eta }},$$

where *I*(*X*_*i*_; *Y*) denotes the MI between the *i*-th genetic factor and the cancer stage, and *η* is a nonnegative constant that regularizes the influence of MIs on the gene selection. When *η* is zero, all variables may be selected with equal probability. Once the hyperedges have been generated, the mean vectors and covariance of the hyperedges are calculated from the training dataset. To identify putative strongly-interacting miRNA-mRNA modules, the initial weight of the *i*-th hyperedge is computed using the variances of each genetic factor and the multivariate MI [65] among all variables, including the class label involved in the hyperedge. A gene with a particular mean expression value but small variance likely possesses higher discriminative capability than one with larger variance. Moreover, by the definition of MI, large multivariate MI implies more relationships among the genes. Thus the initial weight of a hyperedge is defined as

(11) $$w_{0}\left(e_{i|y}\right)=\kappa \cdot I\left(e_{i}\right)+\sum_{x_{\mathrm{ij}}\in e_{i}}\frac{1}{\sigma_{\mathrm{ij}}^{2}{}_{|y}},$$

s.t.

$$\begin{array}{l} I\left(e_{i}\right)=I(X_{i1,};..;X_{\mathrm{ik}};Y)\\ \;=I(X_{i1,};..;X_{\mathrm{ik}})-I(X_{i1,};..;X_{\mathrm{ik}}|Y)\\ \;=I\left(X_{i1,};..;X_{\mathrm{ik}}\right)-E_{Y}(I(X_{i1,};..;X_{\mathrm{ik}})|Y), \end{array}$$

where *k* is the number of variables of *e*_*i*_ and *κ* denotes the ratio of the variance to MI.

**Figure 9:**
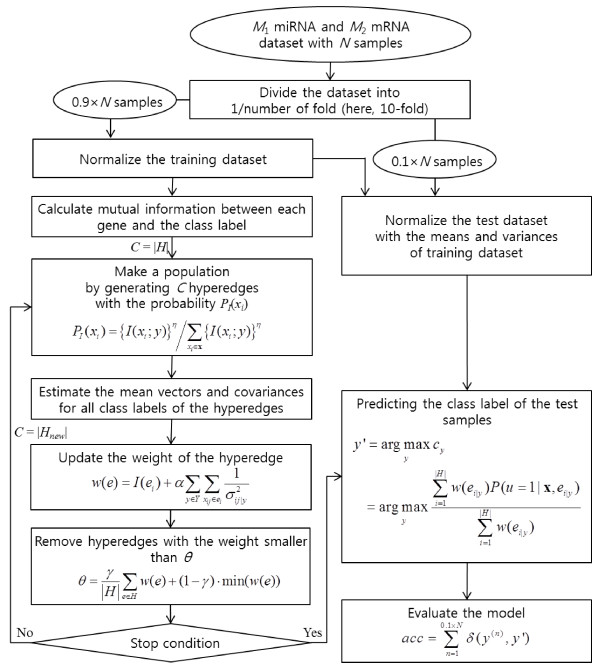
Algorithm for learning the hypergraph-based model.

In the parameter learning phase, the weights of the hyperedges are updated using the gradient descent method for all training data. The aim is to minimize the error in terms of the classification probability in (9) and the matching probability in (5):

(12) $$w_{t}\left(e_{i|y}\right)=\Delta w_{t,i|y}+w_{t-1}\left(e_{i|y}\right),$$

(13) $$\begin{array}{l} \Delta w_{t,i|y}=\frac{\gamma }{t}P\left(y|x,z,H\right)\left(1-P\left(y|x,z,H\right)\right)\\ \;\times \left(\delta \left(\tilde{y},y\right)-P\left(y|x,z,H\right)\right)\cdot P\left(u=1|x,z,e_{i|y}\right), \end{array}$$

where $\tilde{y}$ is the real cancer stage of a miRNA-mRNA expression sample, and *t* and *γ* denote the epoch number in the parameter learning and the parameter learning rate, respectively. The epoch is the number of weight updates for the built hypergraph during parameter learning, and *γ* controls the extent of weight change during parameter learning. Thus, the weight becomes high when the hyperedge consists of miRNAs and mRNAs with strong higher-order interactions and when the variances of the gene variables are small at all cancer stages. Following parameter learning, low weighted hyperedges are removed from the population, and the next structure learning step is performed. To prevent the removal of highly discriminating hyperedges, the number of replaced hyperedges decreases to a specific value as the iterations proceed, as follows:

(14) $$R_{t}=\frac{R_{\max}-R_{\min}}{\exp\left(t\right)}+R_{\min},$$

where *t* is the iteration number of the structure learning phase, and *R*_*max*_ and *R*_*min*_ denote the maximum and minimum number of replaced hyperedges, respectively. Therefore, the number of replaced hyperedges consecutively decreases as the structure learning proceeds, while high-discriminative modules are preserved. The algorithm for learning the hypergraph-based model is presented in Figure 9.

### Representing interaction networks from hypergraphs

**Figure 10:**
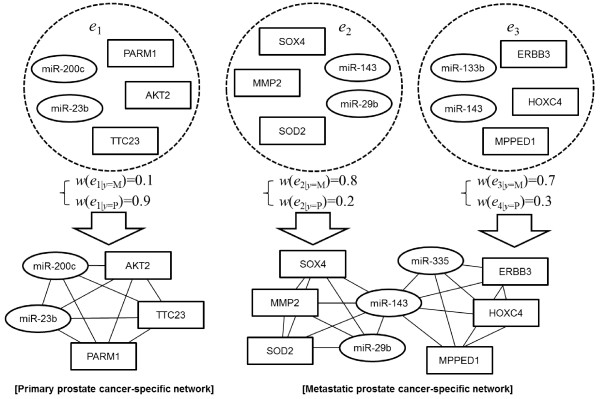
**Procedure of converting a hypergraph to cancer stage-specific interaction networks.** ‘P’ and ‘M’ denote metastatic and primary prostate cancer, respectively.

We construct a higher-order miRNA-mRNA interaction network at a specific cancer stage from the learned model. When analyzing complex biological networks based on graph mining, frequently occurring subgraphs in the networks are generally regarded as important building blocks which are merged to create the functional network [66-69]. Since a high-weight hyperedge corresponds to a significant subgraph reflecting a higher-order relationship among genetic variables, the interaction network is constructed by connecting cliques sharing common genes. A hyperedge is assigned separate weights for each cancer stage and it is merged into the graph of the highest weighted cancer stage. Formally, a cancer-stage *y* ' and a cancer stage-specific interaction network *G*_|*y* '_ =(*V*, *E*), where *V* and *E* denote a vertex set and an edge set, respectively, is constructed by merging the hyperedges as follows (where *y* ' is the class label with the largest weight value):

(15) $$G_{|y'}=G_{|y'}\cup C_{i},$$

(16) $$y'=\underset{y\in Y}{\arg\;\max}\left\{w\left(e_{i|y}\right)\right\},$$

and *C*_*i*_ is a clique corresponding to the *i*-th hyperedge *e*_*i*_ (Figure 10). This dividing and remerging approach enables the constructed interaction networks to be easy-to-visualized without impairing the higher-order property of the model since the weight of edges in the constructed networks are derived from the hyperedge weights reflecting the strength of the higher-order interaction.

## Competing interests

The authors declare that they have no competing interests.

## Authors’ contributions

SJK proposed the idea and wrote the manuscript and analyzed the data. JWH implemented the method and performed the computational experiments. BTZ supervised the study and revised the manuscript. All authors read and approved the final manuscript.
